# Mortality among over 6 million internal and international migrants in Brazil: a study using the 100 Million Brazilian Cohort

**DOI:** 10.1016/j.lana.2023.100455

**Published:** 2023-02-27

**Authors:** Julia M. Pescarini, Emanuelle F. Goes, Priscila Fernanda Porto Scaff Pinto, Beatriz Pinheiro Schindler Dos Santos, Daiane B. Machado, Ibrahim Abubakar, Laura C. Rodrigues, Elizabeth B. Brickley, Liam Smeeth, Mauricio L. Barreto

**Affiliations:** a Faculty of Epidemiology and Population Health, London School of Hygiene & Tropical Medicine, London, UK; b Centre of Data and Knowledge Integration for Health (CIDACS), Gonçalo Moniz Institute, Oswaldo Cruz Foundation, Salvador, Bahia, Brazil; c Instituto de Saúde Coletiva, Universidade Federal da Bahia, Salvador, Brazil; d Department of Global Health and Social Medicine, Harvard Medical School, Boston, MA, USA; e Faculty of Population Health Sciences, University College London (UCL), London, UK; f Health Data Research (HDR), London, UK

**Keywords:** Health inequalities, Disadvantaged groups, Mortality, Maternal health, Migration

## Abstract

**Background:**

To understand if migrants living in poverty in low and middle-income countries (LMICs) have mortality advantages over the non-migrant population, we investigated mortality risk patterns among internal and international migrants in Brazil over their life course.

**Methods:**

We linked socio-economic and mortality data from 1st January 2011 to 31st December 2018 in the 100 Million Brazilian Cohort and calculated all-cause and cause-specific age-standardised mortality rates according to individuals' migration status for men and women. Using Cox regression models, we estimated the age- and sex-adjusted mortality hazard ratios (HR) for internal migrants (i.e., Brazilian-born individuals living in a different Brazilian state than their birth) compared to Brazilian-born non-migrants; and for international migrants (i.e., people born in another country) compared to Brazilian-born individuals.

**Findings:**

The study followed up 45,051,476 individuals, of whom 6,057,814 were internal migrants, and 277,230 were international migrants. Internal migrants had similar all-cause mortality compared to Brazilian non-migrants (aHR = 0.99, 95% CI = 0.98–0.99), marginally higher mortality for ischaemic heart diseases (aHR = 1.04, 95% CI = 1.03–1.05) and higher for stroke (aHR = 1.11, 95% CI = 1.09–1.13). Compared to Brazilian-born individuals, international migrants had 18% lower all-cause mortality (aHR = 0.82, 95% CI = 0.80–0.84), with up to 50% lower mortality from interpersonal violence among men (aHR = 0.50, 95% CI = 0.40–0.64), but higher mortality from avoidable causes related to maternal health (aHR = 2.17, 95% CI = 1.17–4.05).

**Interpretation:**

Although internal migrants had similar all-cause mortality, international migrants had lower all-cause mortality compared to non-migrants. Further investigations using intersectional approaches are warranted to understand the marked variations by migration status, age, and sex for specific causes of death, such as elevated maternal mortality and male lower interpersonal violence-related mortality among international migrants.

**Funding:**

10.13039/100010269The Wellcome Trust.

**Research in context**

**Evidence before this study**

We searched PubMed on 11 January 2022 for studies published since 2010 comparing mortality between migrants and the local-born population using the terms “migrant”, “immigrant”, “refugee”, “emigrant” or “displaced” and “mortality” or “death”. We identified one systematic review evaluating differences in mortality between international migrants and non-migrants; and 18 original studies conducted in low- or middle-income countries (LMICs) examining mortality differences between internal migrants (N = 16), international migrants (N = 1) or between both (N = 1) and the locally-born population. The systematic review analysed studies from 2001 to 2017 and found that mortality among international migrants was lower than the general population but scarce data was available for migrants living in LMICs and only one study from Brazil was included in the metanalysis. Findings from the original studies varied, with some evidence suggesting higher maternal (two out of five studies) and child mortality (three out of six studies) among internal migrants compared to non-migrants among migrants. Studies also reported some evidence of higher maternal mortality (three out of four) among internal migrants compared to non-migrants, and another found higher mortality among international refugees compared to the local-born population. Finally, of the three studies in China analysing specific causes of mortality, two found higher injury-related mortality among internal children and adolescent migrants and one found lower in-hospital HIV mortality among rural-to-urban migrants compared to non-migrants.

**Added value of this study**

To our knowledge, this is the first study to set up a cohort using linked administrative data to study health inequalities between migrants and local-born populations in an LMIC. By analysing data on over 45 million individuals, including over 6 million internal migrants and 277 thousand international migrants followed for up to 15 years, who registered to receive social benefits in Brazil, we observed similar all-cause mortality rates in Brazilian-born internal migrants and non-migrants but lower all-cause mortality among international migrants compared to Brazilian-born individuals. Of public health importance, we found that young international migrant women had higher mortality rates, especially for maternity-related causes than their Brazilian-born counterparts. In comparison, international migrant men had lower violence-related mortality rates than their Brazilian-born counterparts.

**Implications of all the available evidence**

This study provides evidence of an existing ‘healthy migrant effect’ among international migrants living in Brazil that is not consistent for all age and gender groups, nor observed among internal migrants. Differences in mortality rates by age and sex underscore the importance of an intersectional approach to understanding health inequalities in this expanding population group. Further investigation of the disparities in maternal mortality between international migrants and local-born populations may inform local public health policies and reduce preventable causes of death.

## Introduction

It is estimated that over 85 million international migrants and even larger populations of internal migrants, including internally displaced persons, rural-to-urban migrants, and individuals moving along labour corridors, reside in low and middle-income countries (LMICs).^1^ In Latin America and the Caribbean, growing political and economic crises have contributed to the approximate doubling of the number of international migrants over the past two decades.^2^

Current evidence suggests that migrants are generally subjected to higher poverty levels than the local population but, at the same time, are healthier and have overall lower mortality rates than the local population or that of their country of origin (reported as the ‘healthy migrant effect’)^3^^,^^4^ Migrant mortality advantages possibly caused by the selection of individuals that were able to migrate and succeed in the migration journey^5^ depends on the country of origin and is likely to decrease over time and over generations.^6^ However, while there is evidence that the environment in which migrants live can increase their risk of infectious diseases and violence, most of the studies looking at mortality disparities between international migrants and the local-born population are from migrants living in high-income countries (HICs).^3^ Therefore, the extent to which these findings are generalisable across LMIC settings remains uncertain. Compared to their counterparts in HICs, migrants living in LMICs are frequently subjected to higher levels of social deprivation and lower access to health services, and specific policies to recognise migrants as potentially vulnerable groups within LMICs remain rare.^7^

To improve understanding of health disparities in mortality between locally-born and migrant populations in an LMIC setting, we (*i*) investigated the differences in demographic and socio-economic characteristics of internal and international migrants and non-migrants in a cohort of low-income individuals living in Brazil; (*ii*) provided the first estimates of sex-specific age-standardised mortality rates for each group; and (*iii*) investigated the disparities in death risk, considering overall mortality as well as specific causes of death, between internal migrants and Brazilian-born non-migrants and between international migrants and Brazilian-born individuals.

## Methods

### Data sources and data linkage

Our study used nationwide socio-economic and mortality data linked in *The 100 Million Brazilian Cohort* (100MCohort).^8^ The 100MCohort includes baseline demographic and socio-economic data from low-income individuals at their first registration with the Brazilian Unified Registry for Social Programs (*Cadastro Único*, *CadÚnico*). Individuals with per capita income of up to ½ minimum wage (125 USD, considering an approximate exchange rate of 1:4 as in 2018) or familial income of up to 3 minimum wages (750 USD) are eligible to enrol in *CadÚnico*. Still, eligibility to different social programmes (i.e., the conditional cash transfer “Bolsa Familia Programme”, housing or other social benefits) is based on each programme's eligibility criteria.^9^ In addition, to enrol in *CadÚnico*, individuals must provide an official document from the family unit head (≥16 years and preferably a woman), but proof of address is not mandatory.^9^ For this study, from the 100M Cohort baseline, we extracted individual-level information on age, sex, race/ethnicity (self-reported as white, black, “pardo” or mixed, indigenous or Asian ancestry), the place of birth (i.e., in Brazil or another country), education (i.e., literacy and years of formal education), employment, and housing and living conditions (e.g., sanitation, crowding, housing and floor type and material).

Mortality data were extracted from the nationwide Mortality Information System Database (Sistema de Informação sobre Mortalidade, SIM). From SIM we extracted information on the date of death and the primary and secondary causes of death certified by medical professionals and coded according to the International Classification of Diseases version 10 (ICD10).^10^

We linked the 100M Cohort to SIM using five individual level identifiers: name, mother's name, sex, date of birth and municipality of residency. CIDACS-RL, a two-stage linkage algorithm that first uses a deterministic linkage and a second linkage based on a similarity score between the pairs, was used to link the data.^11^ The dataset was generated exclusively for the research aims of this study. After estimating data accuracy, the individual level identifiers were removed from the dataset, and the researcher only had access to the data through a Virtual Private Network (VPN).

### Study population

We included all individuals registered in *CadÚnico* between 1st January 2011 and 31st December 2018. We selected 2011 as the start of follow-up as this was when *CadÚnico* incorporated more accurate questions on the country of birth. We excluded individuals: (*i*) with a death registry before their registration in the 100MCohort (i.e., as a potential sign of linkage error), (*ii*) who were older than 100 years old at enrolment, and (*iii*) whose date of birth was missing. We defined internal migrants as individuals living in a different state than that where they were born and international migrants as those residing in Brazil who had been born in a foreign country.

### Primary and secondary outcomes

The primary outcome was all-cause mortality. Secondary outcomes included common causes of mortality and groups of avoidable causes of death.^12^ The ten highest causes of mortality in Brazil^13^ include ischaemic heart diseases (IHD), stroke, lower respiratory tract infections (LRTI), chronic obstructive pulmonary disease (COPD), interpersonal violence, diabetes, Alzheimer's and other dementias, road injuries, chronic kidney disease and cirrhosis and other chronic liver diseases. Avoidable causes of death include vaccine-preventable and/or treatable infectious diseases (IDs), non-communicable diseases (NCDs), maternal causes, and external and violent causes (see Supplementary Table S1 for ICD-10 codes).

### Analysis

We estimated all-cause and cause-specific age-standardised mortality rates according to individuals' migration status per 100,000 person-years at risk for women and men during the study period. Direct standardisation was applied using the UN World standard population for 2020^14^ and the Brazilian 2020 official population projections^15^; 95% confidence intervals (95% CI) were estimated according to Breslow and Day.^16^ To investigate if being an internal or international migrant was associated with higher mortality risk, we used Cox proportional hazards models to calculate sex-specific age-adjusted mortality hazard ratios (HRs). We compared internal migrants to Brazilian-born non-migrants and international migrants to all Brazilian-born individuals (i.e., independent if they were internal migrants or not). To investigate changes in HRs for all-cause mortality over the life course, we also estimated age-specific HRs. Analyses were performed in Stata 16 and R Studio Version 1.3.1093.

### Ethics

The 100 Million Cohort Study and this study were approved by the ethics committees from Instituto Gonçalo Muniz—Oswaldo Cruz Foundation (Num. 1.612.302 in 2016 and 4.534.397 in 2021). This study was also approved by the ethics committee of The London School of Hygiene & Tropical Medicine. The identified dataset was provided exclusively for this study and further data access requests must be submitted to Cidacs/Fiocruz subject to approval from Oswaldo Cruz Foundation ethical committee.

### Role of the funding source

The funders had no role in the study design, data collection, data analysis, interpretation, or manuscript writing.

## Results

The study included 45,051,476 individuals who applied to *CadÚnico* and were included in the 100MCohort between 2011 and 2018. 44,774,246 (99.4%) individuals were Brazilian-born, of whom 6,057,814 (17.4%) were internal migrants, and 277,230 (0.6%) were international migrants (Supplementary Fig. S1). Notably, 122,122 (44.1%) international migrants joined the 100MCohort in 2018.

Relative to Brazilian-born non-migrants, internal migrants in the 100MCohort were older (median age of 39 (IQR 22–59) vs 14 (1–37) years), had a similar proportion of women (47.0 vs 49.0%), and generally had more favourable indicators of socio-economic position, with lower proportions who had never been to school (19.5 vs 39.5%) and living in overcrowded households (>3 people per room; 23.2 vs 28.2%) and higher proportions living in urban areas (87.8 vs 82.8%) and in households that have surrounding sidewalks (68.1% vs 62.2%) (Table 1, columns 1 and 2).

**Table 1:** Description of study population according to migration status in the 100 Million Brazilian Cohort, 2011–2018.

	Brazilian-born individuals^a^			International migrants (N = 277,230) N (%)
	Internal migrants (N = 6,057,814) N (%)	Non-migrants (N = 28,586,562) N (%)	All Brazilian-born (N = 44,774,246) N (%)	
Age in years (median (IQR))	38.9 (22.0–58.5)	14.2 (1.3–36.9)	17.2 (1.8–39.2)	30.7 (8.6–52.0)
Sex				
Male	2,845,446 (47.0)	14,007,673 (49.0)	21,797,150 (48.7)	144,818 (52.2)
Female	3,212,368 (53.0)	14,578,889 (51.0)	22,977,096 (51.3)	132,412 (47.8)
Race/ethnicity				
White	2,144,879 (36.7)	10,034,611 (35.5)	15,465,863 (35.3)	105,118 (39)
Black	355,147 (6.1)	1,602,999 (5.7)	2,523,242 (5.8)	42,827 (15.9)
Asian	40,892 (0.7)	175,702 (0.6)	281,366 (0.6)	4213 (1.6)
Brown (“Pardo”)	3,292,025 (56.3)	16,211,177 (57.4)	25,236,887 (57.6)	115,689 (42.9)
Indigenous	14,092 (0.2)	210,358 (0.7)	306,409 (0.7)	1692 (0.6)
Missing	210,779	351,715	960,479	7691
Education (higher household education if aged <18)				
Never went to school	1,169,612 (19.5)	11,074,169 (39.5)	16,295,868 (37)	64,655 (23.6)
Primary school or less (≤5 years of education)	2,122,861 (35.4)	7,212,900 (25.7)	12,125,322 (27.5)	83,160 (30.3)
Junior high school (≤9 years of education)	988,916 (16.5)	3,355,990 (12)	5,586,450 (12.7)	40,839 (14.9)
High school or more	1,723,206 (28.7)	6,418,151 (22.9)	10,004,800 (22.7)	85,757 (31.3)
Missing	53,219	525,352	761,806	2819
Work (individuals aged ≥16 years)^b^				
Unemployed/do not work	2,925,515 (62)	8,470,969 (62.6)	13,693,980 (61.8)	117,034 (66.3)
Employed (including internships)	1,720,790 (36.4)	4,705,971 (34.8)	7,862,801 (35.5)	56,753 (32.1)
Retired/pension	75,529 (1.6)	363,188 (2.7)	604,013 (2.7)	2799 (1.6)
Missing	146,150	263,253	761,952	9374
Presence of child labour (<16 y) in the household^c^				
Yes	12,471 (0.6)	750,374 (0.7)	156,062 (0.8)	289 (0.6)
No	2,043,865 (99.4)	10,199,685 (99.3)	20,161,066 (99.2)	52,248 (99.4)
Missing	6233	280,333	48,679	239
Received social benefits from BFP (at any time)				
No	3,468,492 (57.3)	12,609,753 (44.1)	19,103,673 (42.7)	143,490 (51.8)
Yes	2,589,322 (42.7)	15,976,809 (55.9)	25,670,573 (57.3)	133,740 (48.2)
Region of residence				
North	702,594 (11.6)	3,163,368 (11.1)	5,154,036 (11.5)	20,145 (7.3)
Northeast	1,038,531 (17.1)	9,041,939 (31.6)	13,207,506 (29.5)	45,093 (16.3)
Southeast	2,681,972 (44.3)	10,991,723 (38.5)	17,491,152 (39.1)	134,296 (48.4)
South	598,960 (9.9)	3,512,537 (12.3)	5,218,823 (11.7)	54,747 (19.7)
Central-west	1,035,751	1,876,995	3,702,721 (8.3)	22,948 (8.3)
Area of residence				
Urban	5,292,271 (87.8)	23,628,893 (82.8)	37,337,703 (83.5)	248,352 (90.3)
Rural	737,244 (12.2)	4,909,532 (17.2)	7,352,636 (16.5)	26,630 (9.7)
Missing	28,299	48,137	83,907	2248
Household type				
Private and permanent	5,585,075 (94.9)	26,833,783 (95)	41,575,451 (95.1)	254,066 (93.7)
Private but improvised or collective (e.g., tenements)	302,248 (5.1)	1,400,137 (5)	2,143,685 (4.9)	17,154 (6.3)
Missing	170,491	352,642	1,055,110	6010
People per room				
<1.5	1,096,644 (34)	3,439,206 (27)	6,440,477 (26.1)	25,649 (30.6)
1.5–3	1,377,103 (42.7)	5,701,839 (44.8)	10,958,551 (44.4)	36,269 (43.3)
≥3	748,795 (23.2)	3,584,783 (28.2)	7,305,187 (29.6)	21,815 (26.1)
Missing	2,835,272	15,860,734	20,070,031	193,497
Material of the household				
Masonry/brick	5,097,411 (89)	23,622,951 (87)	36,692,703 (86.2)	234,383 (90.9)
Coated or Uncoated Taipa (Rammed earth)	157,323 (2.7)	1,099,895 (4.1)	1,977,660 (4.6)	5515 (2.1)
Wood	472,485 (8.2)	2,415,302 (8.9)	3,875,807 (9.1)	17,925 (7)
Missing	330,595	1,448,414	2,228,076	19,407
Floor material in the household				
No floor (Earth)	324,839 (5.7)	1,510,262 (5.6)	3,211,661 (7.5)	9799 (3.8)
Cement	1,623,343 (28.3)	9,130,352 (33.6)	14,340,431 (33.7)	60,254 (23.4)
Wood	283,492 (4.9)	1,984,591 (7.3)	3,033,801 (7.1)	16,960 (6.6)
Ceramic or stone	3,495,590 (61)	14,513,074 (53.5)	21,960,744 (51.6)	170,815 (66.3)
Missing	330,550	1,448,283	2,227,609	19,402
Sewage disposal				
Public network	3,294,602 (59.4)	14,563,371 (56.7)	23,006,231 (57.1)	172,615 (68.4)
Septic tank	743,697 (13.4)	3,756,801 (14.6)	5,735,274 (14.2)	27,820 (11)
Rudimentary trench	1,346,188 (24.3)	6,390,391 (24.9)	9,930,996 (24.7)	46,676 (18.5)
Others (ditch, river, etc)	160,597 (2.9)	983,399 (3.8)	1,607,963 (4)	5226 (2.1)
Missing	512,730	2,892,600	4,493,782	24,893
Waste disposal/garbage collection				
Collected	5,145,772 (89.8)	23,345,003 (86)	36,452,899 (85.7)	237,954 (92.3)
Burned or buried	485,962 (8.5)	3,274,143 (12.1)	5,092,459 (12)	16,565 (6.4)
Other outdoor disposal	95,488 (1.7)	519,009 (1.9)	1,000,828 (2.4)	3304 (1.3)
Missing	330,592	1,448,407	2,228,060	19,407
Electricity				
Electric with meter	4,905,959 (85.7)	22,658,304 (83.5)	35,284,523 (82.9)	223,182 (86.6)
Electric with community meter	418,702 (7.3)	1,879,767 (6.9)	3,010,161 (7.1)	22,157 (8.6)
Informal electric lights or no electricity	247,438 (4.3)	1,589,590 (5.9)	2,527,994 (5.9)	8278 (3.2)
No electricity	155,121 (2.7)	1,010,495 (3.7)	1,723,532 (4.1)	4206 (1.6)
Missing	330,594	1,448,406	2,228,036	19,407
Bathroom in the household				
Yes	5,533,348 (96.6)	25,653,224 (94.5)	40,137,855 (94.3)	252,033 (97.8)
No	193,877 (3.4)	1,484,923 (5.5)	2,408,340 (5.7)	5790 (2.2)
Missing	330,589	1,448,415	2,228,051	19,407
Sidewalks around the household				
Yes (all around)	3,778,978 (68.1)	16,630,266 (62.2)	25,705,730 (62.4)	187,825 (74.3)
Yes (partially)	337,544 (6.1)	1,695,193 (6.3)	2,642,278 (6.4)	14,057 (5.6)
No	1,435,993 (25.9)	8,395,404 (31.4)	12,824,518 (31.1)	51,015 (20.2)
Missing	505,299	1,865,699	3,601,720	24,333

a 10,129,870 out of 44,774,246 (22.6%) Brazilian-born individuals with missing information for city of birth were only included in the group of all Brazilian-born.

b Calculated for 22,922,746 individuals 16 years or older.

c Calculated for 31,910,152 individuals living in households with at least one child under 16 years in the family.

Relative to all Brazilian-born individuals, international migrants were older (median age of 30.7 vs 17.2 years), had higher proportions of males (52.2% vs 48.7%) as well as those living in the Southeast region (48.4% vs 39.1%) and in urban areas (90.3% vs 83.5%), and generally had more favourable indicators of socio-economic position, with lower proportions who had never been to school (23.6 vs 37.0%) or who were eligible for the *Bolsa Família* conditional cash transfer programme (48.2% vs 57.3%) and higher proportions living in households with covered floors (96.2% vs 92.5%), a public network of sewage disposal (68.4% vs 57.1%), electricity (95.2% vs 90.0%), bathrooms (97.8% vs 94.3%), and at least partially surrounding sidewalks (79.9% vs 68.8%).

**Fig. 1:**
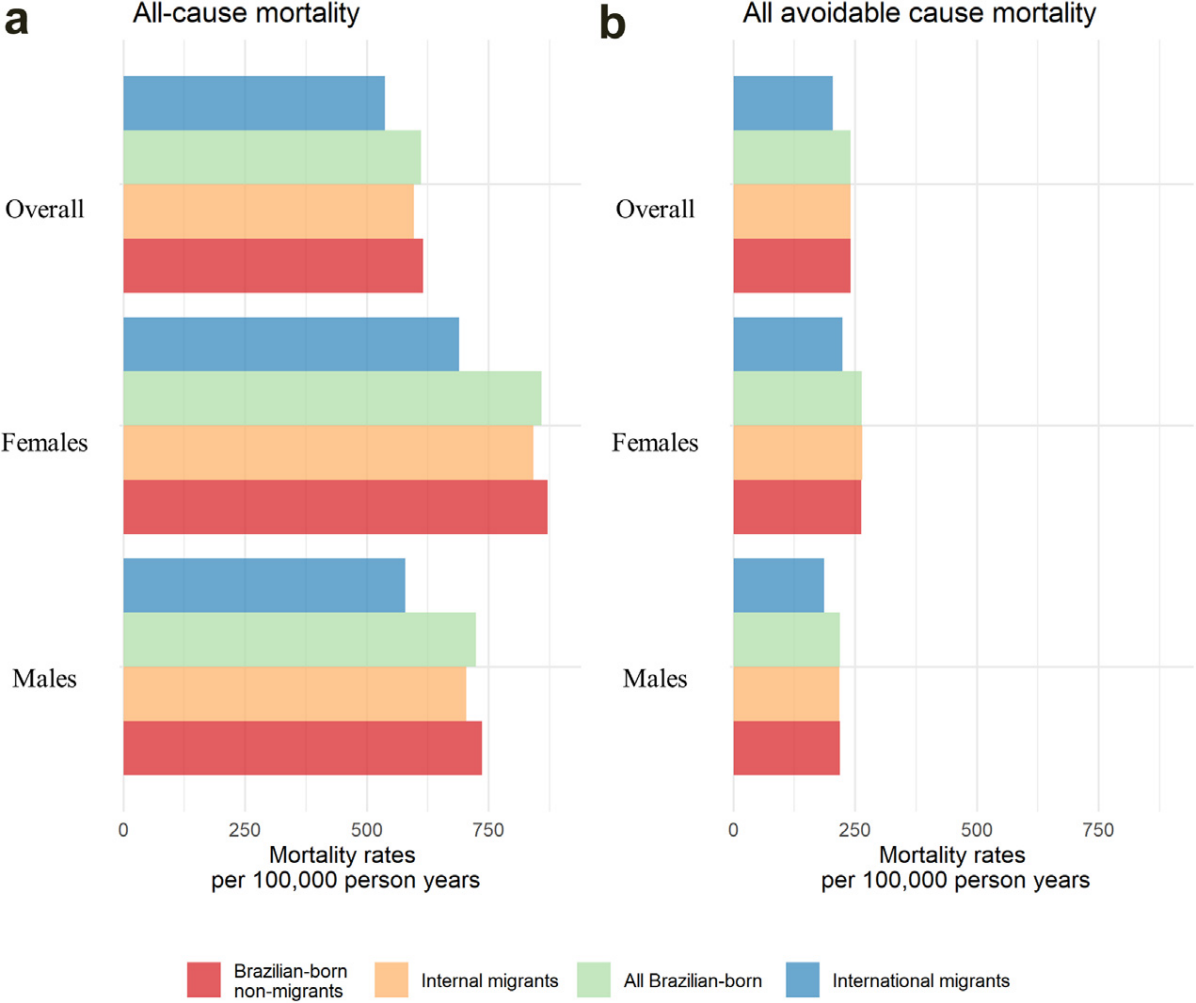
Standardised **(a)** all-cause and **(b)** all avoidable causes mortality rates (SMR) by sex and place of birth among 45,051,476 individuals that applied to social benefit in the 100MCohort from 2011 to 2018.

During the up to 8 years of follow-up (median (IQR) follow-up years: 3 (1.3–4.6) for Brazilian-born non-migrants, 6.0 (4.8–6.8) for internal migrants and 1.6 (0.4–4.5) for international migrants), 778,790 (1.7%) individuals died. The age-standardised mortality rate per 100,000 person-years was 610.3 (95% CI = 610.9–610.9) for the overall Brazilian-born population, which could be broken down as 606.2 (606.2–606.2) for internal migrants and 615.5 (615.5–615.5) for non-migrants, and 536.5 (536.5–536.5) for international migrants (Fig. 1, Supplementary Table S2). All-cause and cause-specific age-standardised mortality rates were generally similar for internal migrants and non-migrants but generally lower for international migrants than Brazilian-born individuals (Supplementary Fig. S3, Tables S2 and S4).

**Fig. 2:**
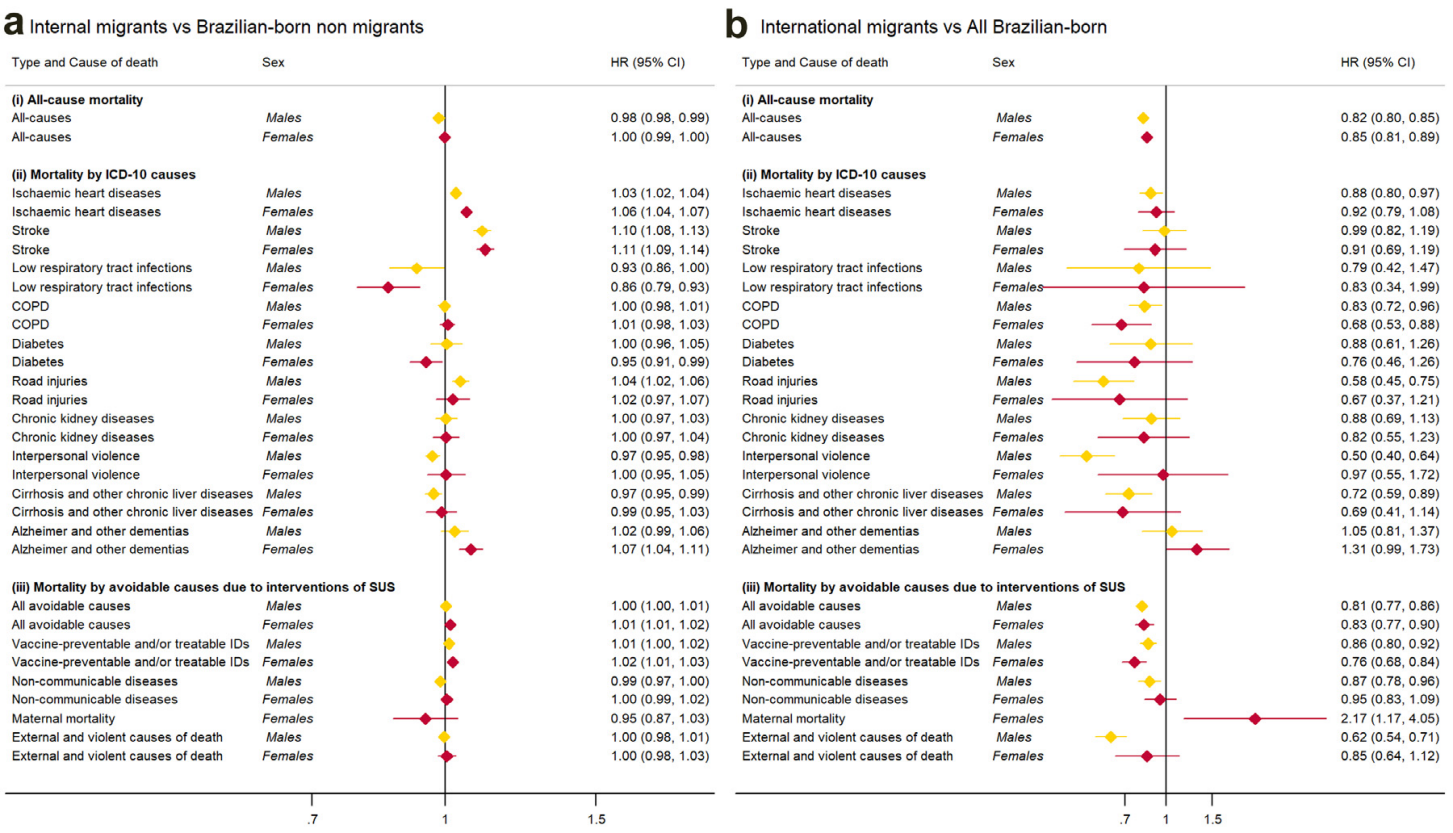
Age-adjusted all-cause and cause-specific mortality hazard ratios (HR) for the association between risk of all-cause death, comparing **(a)** Brazilian-born internal migrants to non-migrants and **(b)** international migrants to all Brazilian-born individuals.

Sex-specific all-cause mortality was similar among internal migrant and non-migrant females (HR = 1.00, 95% CI = 0.99–1.00) but slightly lower among internal migrant men compared to their non-migrant counterparts (HR = 0.98, 95% CI = 0.98–0.99) (see Fig. 2a). Relative to non-migrants, internal migrant females had higher risks of death from IHD (HR = 1.06, 95% CI = 1.04–1.07), stroke (HR = 1.11, 95% CI = 1.09–1.14), and Alzheimer's and other dementias (HR = 1.07, 95% CI = 1.04–1.11) (Supplementary Tables S3 and S5). Relative to non-migrants, male internal migrants had slightly higher risks of death from IHD (HR = 1.03, 95% CI = 1.02–1.04) and stroke (HR = 1.10, 95% CI = 1.08–1.13) but lower risks of death from interpersonal violence (HR = 0.97, 95% CI = 0.95–0.99), and cirrhosis and other chronic liver diseases (HR = 0.97, 95% CI = 0.95–0.99).

International migrants generally had a lower risk of death than their Brazilian-born counterparts (see Fig. 2b, Supplementary Tables S2 and S4). Relative to Brazilian-born women, international migrant women had lower risks of death from all causes (HR = 0.85, 95% CI = 0.81–0.89) as well as from COPD (HR = 0.68, 95% CI = 0.53–0.88), vaccine-preventable and/or treatable IDs mortality (HR = 0.76, 95% CI = 0.68–0.84) but higher risks of maternal death (HR = 2.17, 95% CI = 1.17–4.05) (Supplementary Tables S3 and S5). Relative to Brazilian-born males, international migrants that were male had lower risks of death from all causes (HR = 0.82, 95% CI = 0.80–0.85), IHD (HR = 0.88, 95% CI = 0.80–0.97), COPD (HR = 0.83, 95% CI = 0.72–0.96), road injuries (HR = 0.58, 95% CI = 0.45–0.75), interpersonal violence (HR = 0.50, 95% CI = 0.40–0.64) and cirrhosis and other liver diseases (HR = 0.72, 95% CI = 0.59–0.89). The lower risk of death among international male migrants was also observed for all avoidable causes of death, especially those potentially reduced by intersectoral actions to control violence (HR = 0.62, 95% CI = 0.54–0.71).

**Fig. 3:**
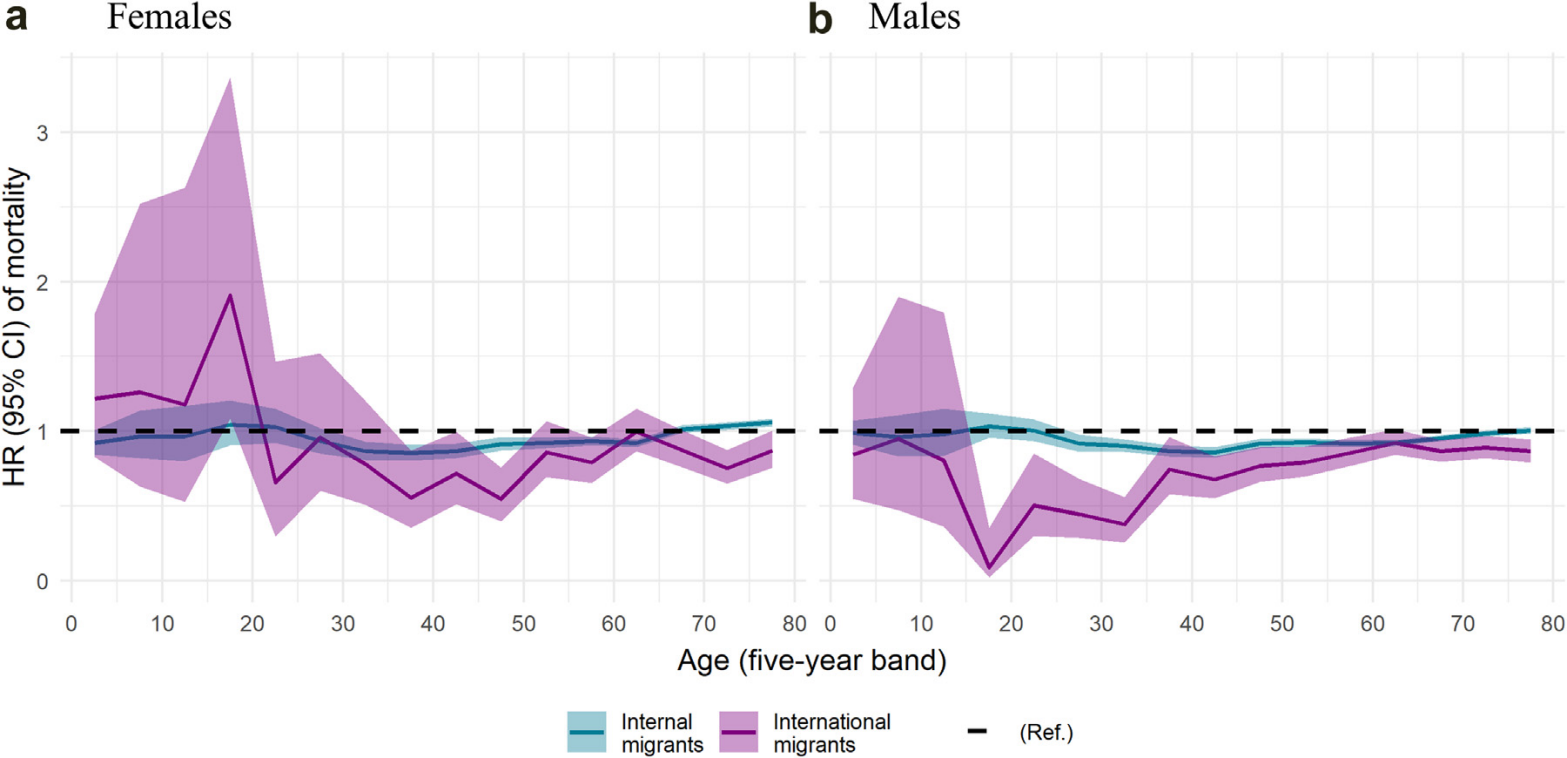
Sex and age-specific mortality hazard ratios (HR) for **(a)** Female and **(b)** Male Brazilian-born internal migrants compared to Brazilian-born non-migrants and for international migrants compared to all Brazilians. The shaded area represents the 95% confidence intervals.

Across the life course, internal migrants have similar sex-specific mortality rates to non-migrants, with slightly lower risks observed among internal migrant adults aged 30–70 years (Fig. 3). Although international migrant males and females generally have lower mortality rates over the different age groups, young international migrant females (i.e., aged 0–20 years) have higher mortality rates than their Brazilian-born counterparts.

## Discussion

In our study, both internal and international migrants were older, with more years of education and living in better conditions than Brazilian born non-migrants. By analysing mortality differences between migrants and non-migrants, our findings provide evidence that internal migration in Brazil was not associated with excess all-cause mortality. Nevertheless, risks varied according to specific causes of mortality, such as elevated stroke and ischaemic heart disease mortality, and by age, with internal migrant adults aged 30–70 years (i.e., reflecting premature mortality) having slight mortality advantages compared to non-migrants. In contrast, international migrants have markedly lower mortality rates than the Brazilian-born population overall, but higher mortality is observed among young females. We also found marked variations by age and sex for specific causes of death, with higher maternal mortality among international migrant assigned as female at birth and a far lower risk of violence-related deaths among male international migrants.

Although non-communicable disease (NCD) mortality is strongly associated with low socio-economic conditions^17^ and largely preventable through timely access to healthcare, previous studies found no consistent differences in all-cause and NCD mortality between internal migrants and non-migrants in LMICs.^18^, ^19^, ^20^, ^21^, ^22^, ^23^ Notably, the studies conducted in Latin America found evidence of lower all-cause mortality among internal and international migrants compared to local-born non-migrants in Peru^20^ and Brazil,^23^ respectively. In contrast to LMICs, several studies have reported mortality advantages among international migrants living in HICs. A 2018 systematic review found that international migrants have 30% lower mortality rates than the general population in the destination countries, with international migrant men having greater mortality advantages than women (28% vs 25%).^3^ Nevertheless, the review included data on seven LMICs (Ethiopia, Guinea, Tanzania, Kenya, Pakistan, Costa Rica and Brazil), for which the only study from Brazil included in their metanalysis was a registry-based study comparing first generation Japanese-Brazilians with local-born Brazilians that found lower all-cause and cancer-related mortality among Japanese-Brazilians.^23^

A 2018 systematic review and meta-analysis of 96 studies with over 15.2 million international migrants to 25 destination countries, of which only seven were classified as an LMIC, demonstrated that international migrants generally have lower all-cause and disease-specific mortality than local-born population groups.^3^ However, they are more likely to die from assaults, infectious diseases and undetermined causes of death.^3^ In our study, international migrants had overall large advantages compared to Brazilians. Nevertheless, we found a lower risk of vaccine-preventable and/or treatable ID mortality. In HIC, international migrants have higher rates of Tuberculosis (TB) than the local-born population, which is usually associated with migrating from a high-burden country for TB and the living conditions in the country of destination.^24^, ^25^, ^26^ Few studies have estimated differences in TB incidence or mortality among international migrants and non-migrants in LMICs.^24^^,^^25^ By investigating mortality differences due to infectious diseases between internal migrants and the local-born population in LMICs, previous studies found suggestive higher tuberculosis mortality among migrant groups in Brazil^24^ but lower HIV related in-hospital mortality in China. Nevertheless, those studies failed to control for important confounders, such as age and HIV-related severity (e.g., CD4 counts), which limit the interpretability of the findings.^24^^,^^27^ Therefore, further analysis should look specifically at important causes of death in migrants, such as TB.

We also found that young migrants (i.e., between 0 and 20 years) have similar or higher mortality than non-migrants compared to slightly older individuals. Internal migrant or internally displaced children have higher under-5 mortality than local-born non-migrant children in many African countries,^28^, ^29^, ^30^ and higher injury-related deaths during childhood and adolescence in China.^31^^,^^32^ Conversely, no consistent differences were found between internal migrant children under-5 and their counterparts in Haiti^33^ and lower under-5 mortality rates were observed for children born to internal migrant mothers in rural Kenya^34^ and South Africa.^35^ In South Africa, children born out of Mozambique international migrants had similar under-5 mortality rates compared to children born to South African mothers.^35^

Higher mortality rates among young international migrant assigned female at birth (i.e., aged 0–20) than Brazilians are likely driven by higher risks of maternal mortality, which was over 2-fold higher among international female migrants in our study. Previous studies in LMIC have found generally higher maternal mortality among internal rural-to-urban^36^^,^^37^ and international^38^ migrants. In China, where access to healthcare is hindered when people move to a region different from where they were originally registered, maternal mortality differences are over 30 times higher among internal migrants than in the local population in Shanghai^37^^,^^39^ and two-times higher among rural migrant women than non-migrants.^36^ In Thailand, a study analysing data on 51 thousand live births in areas of high burden for Malaria found migrant mothers to have a 1.5-fold higher risk of maternal death than non-migrants.^40^ Nevertheless, the only study looking at international migrants in LMICs analysed registry data on live births and maternal deaths from 2010 to 2018 in Lebanon and found Syrian female refugees to have almost two-times higher maternal mortality than Lebanese women in 2018 (17.2 vs 10.2 maternal deaths per 100,000 live births).^38^

A study in Brazil found that Bolivian immigrants have good healthcare utilisation and access to primary healthcare in São Paulo (Brazil), the country's largest urban centre.^41^ Although having access to a universal healthcare system in Brazil can reduce health disparities, maternal deaths mainly occur due to the lack of timely access to prenatal and maternity care during childbirth.^42^, ^43^, ^44^ By analysing the characteristics of Venezuelan migrant women attending sexual and reproductive health services on the Brazilian border before COVID-19 pandemic, 24.0% of pregnant or postpartum women failed to receive any prenatal or postnatal care.^45^ However, qualitative data from the same setting suggests services are generally good despite language barriers and long waiting times.^46^ In the context of migration and increased poverty that can accompany migration, pregnant persons who are migrants face additional barriers in accessing sexual and reproductive healthcare.^42^, ^43^, ^44^ A recent systematic review of over 51 qualitative studies evaluating migrant women's experiences of pregnancy, birth, and maternity care in European countries found migrant women at high risk of poor pregnancy outcomes given the personal and institutional barriers to accessing healthcare services.^42^ In addition, lack of financial resources, support networks, and knowledge of the destination country's health system, along with discrimination and stigma against migrants, can all harm maternal care of pregnant women, increasing the risk of largely preventable deaths.^42^, ^43^, ^44^

Finally, in our study, we found that international male migrants benefit more than females from lower mortality rates related to external causes, both road injuries and interpersonal violence, than their Brazilian counterparts. Homicide rates in Brazil are among the highest globally, with young Black men, with few years of education at disproportionately high risk.^47^^,^^48^ As a significantly higher burden is related to motorcycle accidents in large urban centres,^48^ one possible explanation is that international migrants are living under better socio-economic circumstances and consequently, in better neighbourhoods, may be less likely to die from traffic-related accidents.

To our knowledge, this is the first study to use linked administrative data to understand the health status of international and internal migrants in Brazil or any other LMIC. The cohort's incomparable size enabled us to estimate sex, age, and cause-specific analysis mortality rates among internal and international migrants living in poverty or extreme poverty in Brazil and found important differences from their non-migrant counterparts. Nevertheless, our study is subject to limitations of using a cohort of linked administrative data. First, mortality in very small children (i.e., neonatal death) could have been underestimated as children must be first registered in *CadÚnico* to be linked. Second, we could not measure the loss of follow-up due to emigration from Brazil, which could lead to underestimating the number of deaths. Third, underreporting of deaths might be more likely among international migrants, as sick and older people may move back to their country of origin once labour migrants might not be able to send money back to their country of origin anymore. Fourth, variables used in data linkage could be more likely to be misspelled or to have incomplete information (e.g., on name, name of the mother) in the case of international migrants than locals, possibly resulting in misclassification of deaths and underestimation of mortality among migrants (i.e., leading to a more extreme protective HR for mortality).

Although migrants legally living in Brazil have the right to register in CadUnico and access to social protection policies, registries of migrant families have markedly increased during the study period in two moments. First, in 2014, due to an official note reinforcing the rights of migrants living in poverty in Brazil and the importance for them to access social benefits such as BFP^49^ and later, in 2017 and 2018, due to “Operação acolhida” a governmental programe that facilitated the access to documents and interiorisation of Venezuelan migrants in Brazil.^50^ It is also noteworthy that in this study, we only analysed migrants registered with the 100MCohort and, therefore, we estimated mortality among a low-income population (i.e., not considering wealthy migrants)—wealthier migrants, irregular migrants, recently arrived groups, and those facing extreme poverty could either not be eligible or have difficulties registering in *CadÚnico*. Therefore, we are likely to underestimate health disparities among those groups. Also, we could not estimate the country of origin, time and reason for migration for each individual, which is key to understanding the socio-economic and political context where migrants come from and potential changes in their health status after migration to Brazil.

In 2010 the number of internal migrants in Brazil (i.e., those living in different states than they were born) was estimated to be 14.5% (26.3 million),^51^ but the number of international migrants is largely underestimated—in the same year, Brazilian Home Office estimated, just for the municipality of Sao Paulo, a 10-times higher number of Bolivian migrants^52^ than the Census estimates^53^ for the entire country. From 2010 onwards Brazil observed increased south–south (i.e., from other LMICs) and regional (i.e., from other latin-american countries) migration flows due to natural disasters, economical and political crisis in Latin America.^53^^,^^54^ From 2010 to 2019, 660.349 international migrants were registered as staying at least 1 year in the country, the majority of which from Venezuela (142,250), followed by Paraguai (97,316), Bolívia (57,765) and Haiti (54,182).^55^ In 2020, Venezuelans constituted the second largest population of people displaced across borders globally, with approximately 85% of the 5.6 million Venezuelans that left the country moving to other countries in Latin America.^1^

Although our goal was not to investigate the differences between internal and international migrants, it is noteworthy that first-generation migrants may not be subject to the same drivers of health disparities as internal migrants or children of migrant parents. In addition, as most of the internal migrants in Brazil are labour migrants, they might not be subject to the same level of vulnerability as international migrants or internally displaced populations in other LMICs (e.g., migration due to conflicts or natural disasters)^56^–international migrants may be systematically different from Brazilian-born individuals not just in age and socio-economic characteristics but also in the prevalence of comorbidities and other residual confounders. Nevertheless, as socio-economic circumstances would be in the causal pathway between migration and higher mortality rates, we have most likely provided the best estimate that reflects those differences.

## Conclusions

By investigating health disparities between migrants and non-migrants registered within the 100 Million Brazilian cohort, our study pointed out a disproportionally higher non-communicable disease mortality among internal migrants, and higher maternal mortality but lower violence mortality among international migrants. Gender and age variations in mortality risks should lead to further investigation of the main causes of child mortality among migrants, differences in child mortality according to migration status of the parents, as well as the underlying risk factors associated with cause-specific morbidity, access to healthcare and mortality among migrant mothers to inform the targeting of social and health interventions in LMICs. Tackling the socio-economic determinants of mortality is key as the escalation of the economic crisis in the past ten years, and the COVID-19 pandemic made the living conditions of migrant populations living in Latin American countries even more precarious and has revived the call for a better understanding of their health needs in the context of LMICs.^57^

## Contributors

J.M.P., D.B.M., E.B.B., L.S. and M.L.B. contributed to study conceptualisation, J.M.P., P.S. and B.S. contributed to data curation, J.M.P., E.F.G. and B.S. performed the formal analysis, M.L.B., L.C.R. and L.S. contributed to funding acquisition, J.M.P., E.F.G., P.S., D.B.M., I.A., L.C.R., E.B.B., L.S. and M.L.B. contributed to investigation, J.M.P., E.F.G., D.B.M., L.C.R. and E.B.B. contributed to methodology, E.B.B., L.M. and M.L.B. administrated the project administration, P.S. and B.S. validated the analysis, J.M.P. and B.S. contributed to visualisation, J.M.P., E.F.G., P.S. and D.B.M. contributed to writing the original draft, and all authors contributed to reviewing & editing the final manuscript.

## Data sharing statement

The data underlying this article will be shared on reasonable request to CIDACS Fiocruz and after ethical approval.

## Declaration of interests

We declare no competing interests.

## Appendix A Supplementary data

Supplementary Figures S1–S3 and Tables S1–S6

Translated Summary
