# Supplementary material for: Mortality among over 6 million internal and international migrants in Brazil: a study using the 100 Million Brazilian Cohort
Source: Lancet Reg Health Am. 2023 Feb 27;20:100455. doi: 10.1016/j.lana.2023.100455 (PMC9986634; doi:10.1016/j.lana.2023.100455)
Supplement: Supplementary Figures S1–S3 and Tables S1–S6 [file mmc1.docx]

Supplementary material

**
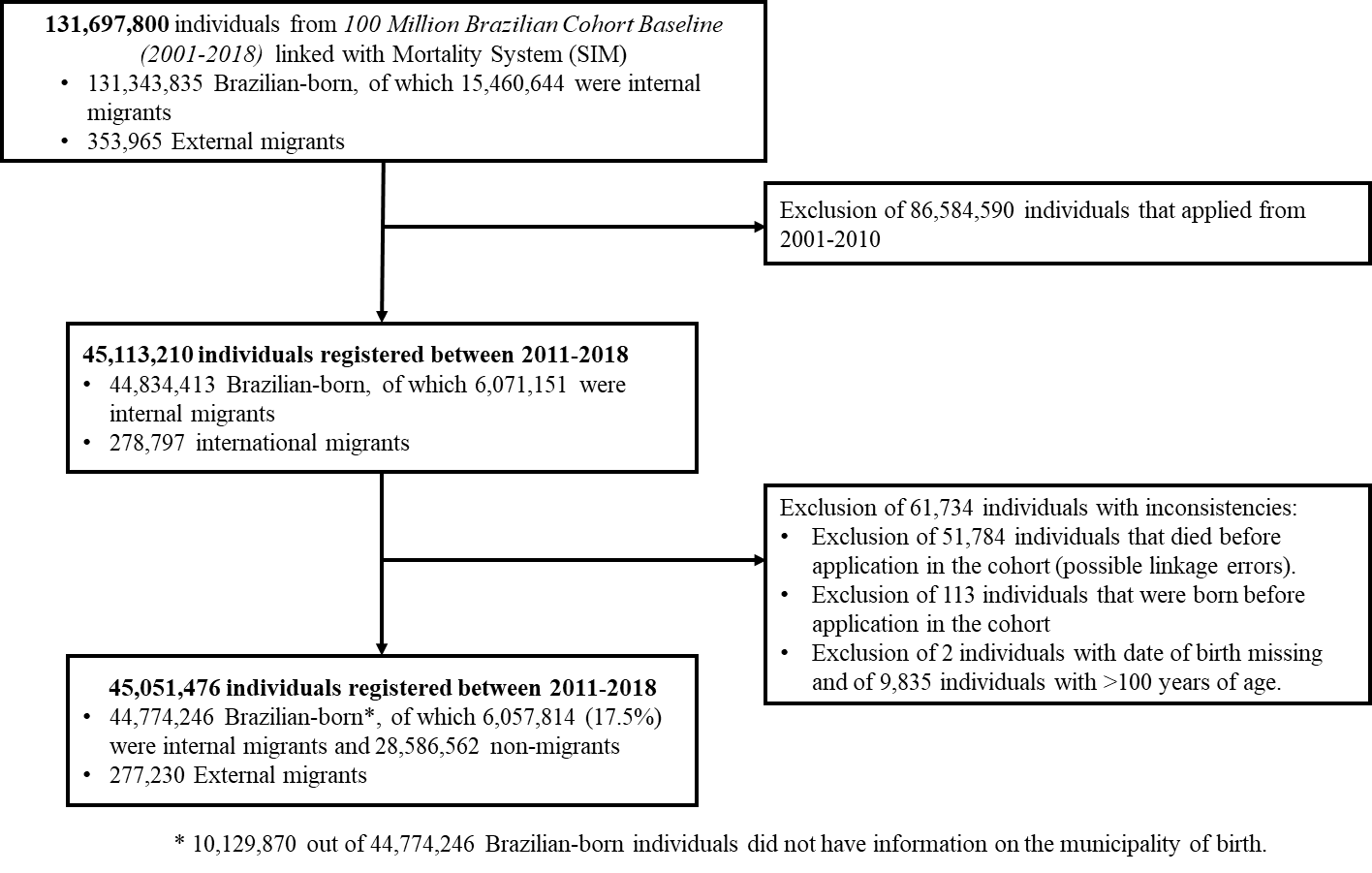
**

**Figure S1.** Flowchart of the study population.

**
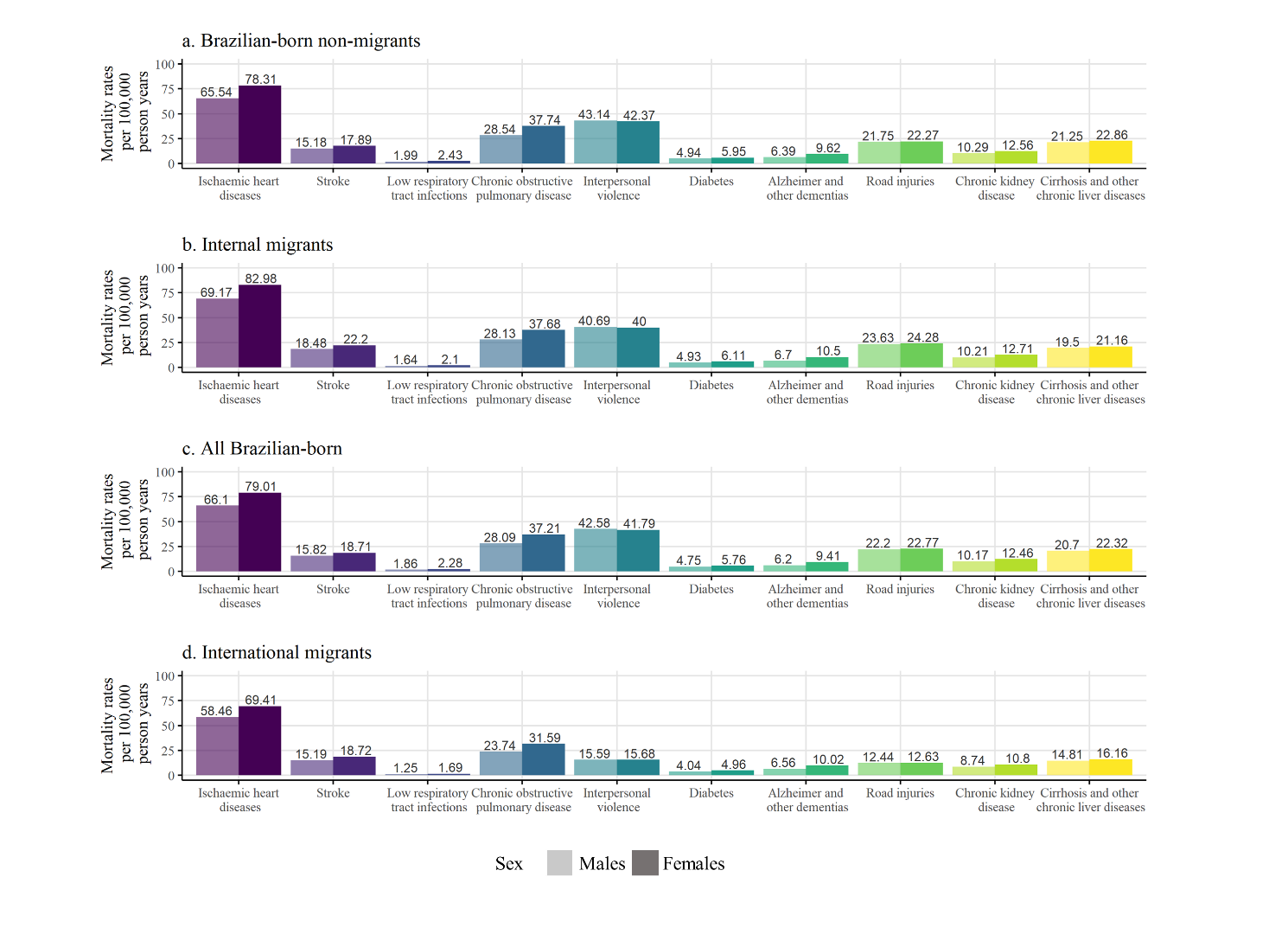
**

**Figure S2.** Age-standardised mortality rates per 100,000 person-years at risk for the 10^th^ highest causes of death in Brazil among Brazilian born individuals who are migrants or not and among non-Brazilian born individuals.


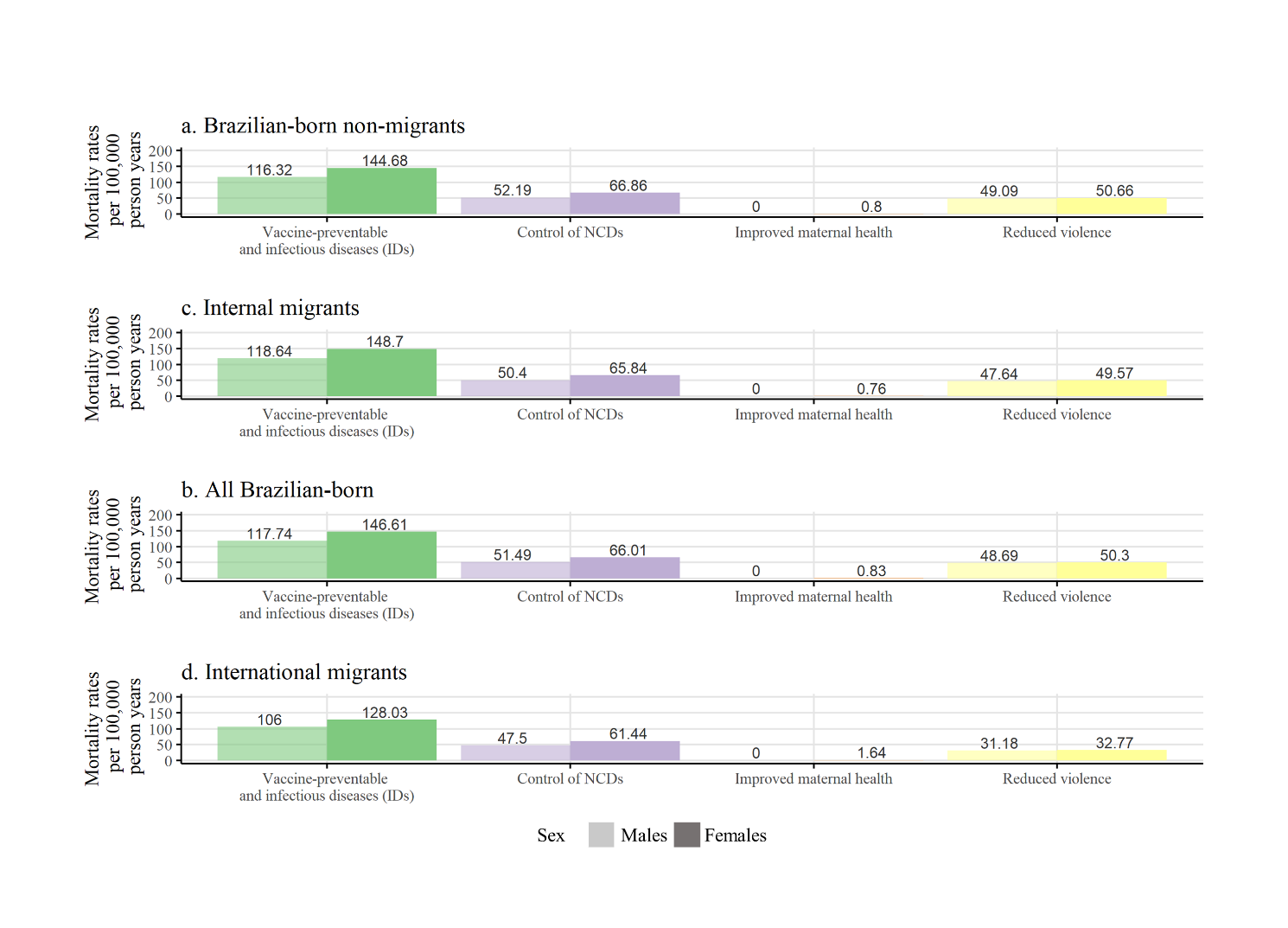


**Figure S3.** Age-standardised mortality rates per 100 000 person-years at risk for avoidable causes of death in Brazil among Brazilian born individuals who are migrants or not, and among non-Brazilian born individuals.

**Table S1· Studied causes of death**

| GBD 10th higher causes of death in Brazil ^56^ | 1. Ischaemic heart diseases (ICD-10, I20-I25·9) 2. Stroke (G45-G46·8, I60-I63·9, I65-I66·9, I67·0-I67·3, I67·5-I67·6, I68·1-I68·2, I69·0-I69·3) 3. Low respiratory tract infections (A48·1, A70, B97·4-B97·6, J09-J15·8, J16-J16·9, J20-J21·9, J91·0, P23·0-P23·4, U04- U04·9) 4. Chronic obstructive pulmonary disease (COPD) (J41-J44·9) 5. Interpersonal violence (X85-Y08·9, Y87·1) 6. Diabetes (E10-E10·1, E10·3-E11·1, E11·3-E11·9, P70·2) 7. Alzheimer and other dementias (F00-F02·0, F02·8-F03·9, G30-G31·1, G31·8-G31·9) 8. Road injuries (V01-V04·9, V06-V80·9, V82-V82·9, V87·2-V87·3) 9. Chronic kidney disease (D63·1, E10·2, E11·2, I12-I13·9, N02-N08·8, N15·0, N18-N18·9, Q61-Q62·8) 10. Cirrhosis and other chronic liver diseases (B18-B18·9, I85-I85·9, I98·2, K70-K70·3, K71·7, K73-K75, K75·2, K75·4-K76·2, K76·4-K76·9, K77·8) |
| --- | --- |
| Avoidable causes of deaths due to interventions of the Brazilian Universal Health System (SUS)^12^ | 1. Vaccine preventable and/or treatable infectious diseases: A15-A19; B90; A34; A35; A36; A37; A80; B05; B06; B16; G00·0·; B57·2;C00; C43-C44; C22; C16; C18-C21; C01-C06; C09; C10; C12-C14; C32; C15; C33; C34; C50; C53-C55; C62; C73; C81; C91; E01-E05; E00; E25·0; E70·0; E74·2; E10-E14; E40-E46; E50-E64; D50-D53; E86; F10; I426; K292; K70; I85; G40; G41; I10-I13; I20-I25; I70; I50; I61; I630-I635; I638; I639; I64-I66; J40-J43; J45-J46; K25-K28; K35; J60-J70; K40-K46; K56; K80-K83; N18 2. Reduced by health promotion and control of non-communicable diseases: A00-A09; B20-B24;B15;B17-B19; A50-A59; A63-A64; N70-N735; N73·8-N73·9; N75; N76; A23-A26; A28-A32; A38; A39; A40; A41; A46; A69·2; J020; J030; B50-B54; G00·1-G00·9; G01; I00-I09; J00; J01; J02·8; J02·9; J03·8; J03·9; J04; J05; J06·0; J10-J22; L02-L08; A20; A21; A22; A27; A30; A77; A82; A90; A92·3; A95; A98·5; B03; B55; B57·0; B57·1; B65; N39·0 3. Reduced by actions to control the causes of maternal mortality: O00-O02; O03-O26; O29-O99 4. Reduced by intersectoral actions to external and violent causes of death: V01-V89; W65-W74; X00-X09; X40-X49; X60-X84; X85-Y09; Y10-Y34; W00-W19; Y60-Y69; Y83-Y84 |

**Table S2.** Age-adjusted mortality rate overall based on the UN and the Brazilian population for 2020, according to the place of birth.

| **Group** | **Std rates (95%CI) using Brazil’s 2020 standard population** | **Std rates (95%CI) using UN 2020 standard population** |
| --- | --- | --- |
| **All-cause mortality** |  |  |
| Brazilian-born | 648·9 (648·9-648·9) | 610·9 (610·9-610·9) |
| Brazilian-born non-migrants | 654·5 (654·5-654·5) | 615·5 (615·5-615·5) |
| Internal migrants | 632·4 (632·4-632·4) | 596·8 (596·8-596·8) |
| International migrants | 573·8 (573·8-573·8) | 536·5 (536·5-536·5) |
| **Ischaemic heart diseases** |  |  |
| Brazilian-born | 57·5 (57·5-57·5) | 54 (54-54) |
| Brazilian-born non-migrants | 56·3 (56·3-56·3) | 52·7 (52·7-52·7) |
| Internal migrants | 60·6 (60·6-60·6) | 56·9 (56·9-56·9) |
| International migrants | 56 (56-56) | 52·5 (52·5-52·5) |
| **Stroke** |  |  |
| Brazilian-born | 16·4 (16·4-16·4) | 15·3 (15·3-15·3) |
| Brazilian-born non-migrants | 15·6 (15·6-15·6) | 14·6 (14·6-14·6) |
| Internal migrants | 19·2 (19·2-19·2) | 18 (18-18) |
| International migrants | 15·9 (15·9-15·9) | 14·8 (14·8-14·8) |
| **Low respiratory tract infections** |  |  |
| Brazilian-born | 1·8 (1·8-1·8) | 1·7 (1·7-1·7) |
| Brazilian-born non-migrants | 2 (2-2) | 1·9 (1·9-1·9) |
| Internal migrants | 1·5 (1·5-1·5) | 1·5 (1·5-1·5) |
| International migrants | 1·5 (1·5-1·5) | 1·4 (1·4-1·4) |
| **Chronic obstructive pulmonary disease (COPD)** |  |  |
| Brazilian-born | 26·6 (26·6-26·6) | 24·9 (24·9-24·9) |
| Brazilian-born non-migrants | 26·9 (26·9-26·9) | 25·1 (25·1-25·1) |
| Internal migrants | 27·2 (27·2-27·2) | 25·5 (25·5-25·5) |
| International migrants | 23·4 (23·4-23·4) | 21·7 (21·7-21·7) |
| **Interpersonal violence** |  |  |
| Brazilian-born | 22·2 (22·2-22·2) | 21·1 (21·1-21·1) |
| Brazilian-born non-migrants | 22·7 (22·7-22·7) | 21·5 (21·5-21·5) |
| Internal migrants | 20·7 (20·7-20·7) | 19·7 (19·7-19·7) |
| International migrants | 10·8 (10·8-10·8) | 10·1 (10·1-10·1) |
| **Diabetes** |  |  |
| Brazilian-born | 5·2 (5·2-5·2) | 4·8 (4·8-4·8) |
| Brazilian-born non-migrants | 5·4 (5·4-5·4) | 5 (5-5) |
| Internal migrants | 5·1 (5·1-5·1) | 4·8 (4·8-4·8) |
| International migrants | 4·2 (4·2-4·2) | 4 (4-4) |
| **Alzheimer and other dementias** |  |  |
| Brazilian-born | 8·5 (8·5-8·5) | 7·9 (7·9-7·9) |
| Brazilian-born non-migrants | 8·6 (8·6-8·6) | 8 (8-8) |
| Internal migrants | 9·4 (9·4-9·4) | 8·9 (8·9-8·9) |
| International migrants | 10·2 (10·2-10·2) | 9·1 (9·1-9·1) |
| **Road injuries** |  |  |
| Brazilian-born | 13·6 (13·6-13·6) | 12·9 (12·9-12·9) |
| Brazilian-born non-migrants | 13·5 (13·5-13·5) | 12·7 (12·7-12·7) |
| Internal migrants | 14·1 (14·1-14·1) | 13·4 (13·4-13·4) |
| International migrants | 9 (9-9) | 8·5 (8·5-8·5) |
| **Chronic kidney disease** |  |  |
| Brazilian-born | 9·4 (9·4-9·4) | 8·8 (8·8-8·8) |
| Brazilian-born non-migrants | 9·4 (9·4-9·4) | 8·8 (8·8-8·8) |
| Internal migrants | 9·4 (9·4-9·4) | 8·9 (8·9-8·9) |
| International migrants | 8·5 (8·5-8·5) | 8 (8-8) |
| **Cirrhosis and other chronic liver diseases** |  |  |
| Brazilian-born | 13·8 (13·8-13·8) | 12·9 (12·9-12·9) |
| Brazilian-born non-migrants | 14 (14-14) | 13·2 (13·2-13·2) |
| Internal migrants | 12·8 (12·8-12·8) | 12·1 (12·1-12·1) |
| International migrants | 11·5 (11·5-11·5) | 10·8 (10·8-10·8) |

**Table S3.** Person years at risk, number of events and age- and sex-adjusted Hazard ratios by cause of death.

| **Subgroups** | **Person years at risk** | **Events** | **HR (95%CI)^1^** |
| --- | --- | --- | --- |
| **All cause mortality** | **pyr=177,295,321** | **778,790** |  |
| Brazilian-born non-migrants | 90 229 761·7 | 3 926 23 | 1 |
| Internal migrants | 23 009 063·1 | 187 397 | 0·99 (0·98-0·99) |
| Brazilian born (all) | 176 599 628·5 | 773 178 | 1 |
| International migrants | 695 692·7 | 5 612 | 0·82 (0·8-0·84) |
| **Ischaemic heart diseases** |  |  |  |
| Brazilian-born non-migrants | 90 229 761·7 | 32 023 | 1 |
| Internal migrants | 23 009 063·1 | 18 994 | 1·04 (1·03-1·05) |
| Brazilian born (all) | 176 599 628·5 | 65 938 | 1 |
| International migrants | 695 692·7 | 571 | 0·88 (0·81-0·96) |
| **Stroke** |  |  |  |
| Brazilian-born non-migrants | 90 229 761·7 | 8 868 | 1 |
| Internal migrants | 23 009 063·1 | 5 815 | 1·11 (1·09-1·13) |
| Brazilian born (all) | 176 599 628·5 | 18 761 | 1 |
| International migrants | 695 692·7 | 161 | 0·96 (0·82-1·12) |
| **Low respiratory tract infections** |  |  |  |
| Brazilian-born non-migrants | 90 229 761·7 | 1 452 | 1 |
| Internal migrants | 23 009 063·1 | 444 | 0·89 (0·85-0·95) |
| Brazilian born (all) | 176 599 628·5 | 2 469 | 1 |
| International migrants | 695 692·7 | 15 | 0·80 (0·48-1·33) |
| **Chronic obstructive pulmonary disease (COPD)** |  |  |  |
| Brazilian-born non-migrants | 90 229 761·7 | 15 448 | 1 |
| Internal migrants | 23 009 063·1 | 8 552 | 1·00 (0·99-1,01) |
| Brazilian born (all) | 176 599 628·5 | 30 470 | 1 |
| International migrants | 695 692·7 | 245 | 0·79 (0·70-0·90) |
| **Interpersonal violence** |  |  |  |
| Brazilian-born non-migrants | 90 229 761·7 | 14 017 | 1 |
| Internal migrants | 23 009 063·1 | 4 319 | 0·97 (0·95-0·99) |
| Brazilian born (all) | 176 599 628·5 | 27 717 | 1 |
| International migrants | 695 692·7 | 79 | 0·54 (0·43-0·68) |
| **Diabetes** |  |  |  |
| Brazilian-born non-migrants | 90 229 761·7 | 3 080 | 1 |
| Internal migrants | 23 009 063·1 | 1 603 | 0·97 (0·95-1·97) |
| Brazilian born (all) | 176 599 628·5 | 5 957 | 1 |
| International migrants | 695 692·7 | 45 | 0·83 (0·62-1·11) |
| **Alzheimer and other dementias** |  |  |  |
| Brazilian-born non-migrants | 90 229 761·7 | 4 724 | 1 |
| Internal migrants | 23 009 063·1 | 2 738 | 1·05 (1·03-1·08) |
| Brazilian born (all) | 176 599 628·5 | 9 146 | 1 |
| International migrants | 695 692·7 | 107 | 1·15 (0·95-1·39) |
| **Road injuries** |  |  |  |
| Brazilian-born non-migrants | 90 229 761·7 | 8 526 | 1 |
| Internal migrants | 23 009 063·1 | 3 489 | 1·03 (1·01-1·06) |
| Brazilian born (all) | 176 599 628·5 | 17 442 | 1 |
| International migrants | 695 692·7 | 68 | 0·60 (0·47-0·76) |
| **Chronic kidney disease** |  |  |  |
| Brazilian-born non-migrants | 90 229 761·7 | 5 394 | 1 |
| Internal migrants | 23 009 063·1 | 2 874 | 1·00 (0·98-1·03) |
| Brazilian born (all) | 176 599 628·5 | 10 807 | 1 |
| International migrants | 695 692·7 | 88 | 0·87 (0·70-1·07) |
| **Cirrhosis and other chronic liver diseases** |  |  |  |
| Brazilian-born non-migrants | 90 229 761·7 | 7 770 | 1 |
| Internal migrants | 23 009 063·1 | 3 769 | 0·97 (0·95-0·99) |
| Brazilian born (all) | 176 599 628·5 | 15 528 | 1 |
| International migrants | 695 692·7 | 106 | 0·71 (0·59-0·86) |

^1^ Age and sex-adjusted mortality hazard rates.

**Table S4.** Age-adjusted preventable mortality rate based on the UN and the Brazilian population for 2020, according to the place of birth.

| **Group** | **Std rates (95%CI) using Brazil’s 2020 standard population** | **Std rates (95%CI) using UN 2020 standard population** |
| --- | --- | --- |
| **All avoidable causes** |  |  |
| Brazilian-born | 255·6 (255·6-255·6) | 240·5 (240·5-240·5) |
| Brazilian-born non-migrants | 255·4 (255·4-255·4) | 240 (240-240) |
| Internal migrants | 254·7 (254·7-254·7) | 240·4 (240·4-240·4) |
| International migrants | 219·6 (219·6-219·6) | 204·3 (204·3-204·3) |
| **Vaccine-preventable** |  |  |
| Brazilian-born | 2·8 (2·8-2·8) | 2·6 (2·6-2·6) |
| Brazilian-born non-migrants | 2·9 (2·9-2·9) | 2·7 (2·7-2·7) |
| Internal migrants | 2·3 (2·3-2·3) | 2·2 (2·2-2·2) |
| International migrants | 3·7 (3·7-3·7) | 3·4 (3·4-3·4) |
| **Non-communicable diseases (NCDs)** |  |  |
| Brazilian-born | 137·9 (137·9-137·9) | 129·3 (129·3-129·3) |
| Brazilian-born non-migrants | 136·2 (136·2-136·2) | 127·6 (127·6-127·6) |
| Internal migrants | 139·7 (139·7-139·7) | 131·3 (131·3-131·3) |
| International migrants | 121·2 (121·2-121·2) | 113·4 (113·4-113·4) |
| **Infectious diseases (IDs)** |  |  |
| Brazilian-born | 62·2 (62·2-62·2) | 58·6 (58·6-58·6) |
| Brazilian-born non-migrants | 63·2 (63·2-63·2) | 59·4 (59·4-59·4) |
| Internal migrants | 61·3 (61·3-61·3) | 58 (58-58) |
| International migrants | 59·5 (59·5-59·5) | 54·4 (54·4-54·4) |
| **Maternal mortality** |  |  |
| Brazilian-born | 0·9 (0·9-0·9) | 0·8 (0·8-0·8) |
| Brazilian-born non-migrants | 0·9 (0·9-0·9) | 0·8 (0·8-0·8) |
| Internal migrants | 0·8 (0·8-0·8) | 0·8 (0·8-0·8) |
| International migrants | 1·8 (1·8-1·8) | 1·7 (1·7-1·7) |
| **External and violent causes of death** |  |  |
| Brazilian-born | 52·1 (52·1-52·1) | 49·5 (49·5-49·5) |
| Brazilian-born non-migrants | 52·7 (52·7-52·7) | 49·8 (49·8-49·8) |
| Internal migrants | 51·1 (51·1-51·1) | 48·6 (48·6-48·6) |
| International migrants | 33·9 (33·9-33·9) | 31·9 (31·9-31·9) |

**Table S5.** Person years at risk, number of events and age- and sex-adjusted Hazard ratios by avoidable causes of death.

| **Subgroups** | **Person years at risk** | **Events** | **HR (95%CI)^1^** |
| --- | --- | --- | --- |
| **All avoidable causes** | **pyr=177,295,321** | **304826** |  |
| Brazilian-born non-migrants | 90 229 761·7 | 151 723 | 1 |
| Internal migrants | 23 009 063·1 | 72 795 | 1·00 (1·00-1·01) |
| Brazilian born (all) | 176 599 628·5 | 302 717 | 1 |
| International migrants | 695 692·7 | 2 109 | 0·80 (0·76-0·83) |
| **Vaccine-preventable and infectious diseases (IDs)** |  |  |  |
| Brazilian-born non-migrants | 90 229 761·7 | 79 540 | 1 |
| Internal migrants | 23 009 063·1 | 43 814 | 1·01 (1·01-1·02) |
| Brazilian born (all) | 176 599 628·5 | 81 835 | 1 |
| International migrants | 695 692·7 | 1257 | 0·82 (0·78-0·87) |
| **Non-communicable diseases (NCDs)** |  |  |  |
| Brazilian-born non-migrants | 90 229 761·7 | 38 889 | 1 |
| Internal migrants | 23 009 063·1 | 17 923 | 0·99 (0·98-1·00) |
| Brazilian born (all) | 176 599 628·5 | 74 898 | 1 |
| International migrants | 695 692·7 | 581 | 0·89 (0·82-0·96) |
| **Maternal mortality** |  |  |  |
| Brazilian-born non-migrants | 90 229 761·7 | 555 | 1 |
| Internal migrants | 23 009 063·1 | 170 | 0·95 (0·87-1·04) |
| Brazilian born (all) | 176 599 628·5 | 1 161 | 1 |
| International migrants | 695 692·7 | 10 | 2·16 (1·16-1·03) |
| **External and violent causes of death** |  |  |  |
| Brazilian-born non-migrants | 90 229 761·7 | 32 991 | 1 |
| Internal migrants | 23 009 063·1 | 12 009 | 0·99 (0·98-1·00) |
| Brazilian born (all) | 176 599 628·5 | 65 786 | 1 |
| International migrants | 695 692·7 | 266 | 0·64 (0·57-0·72) |

^1^ Age and sex-adjusted mortality hazard rates

**Table S6.** Search strategy for developing the research in context session.

|  | **Search** | **11/01/2022** |
| --- | --- | --- |
| #1 | (("migrant"[Title/Abstract]) OR ("immigrant"[Title/Abstract]) OR ("refugee"[Title/Abstract]) OR ("emigrant"[Title/Abstract]) OR ("displaced"[Title/Abstract])) AND ((("mortality"[Title/Abstract]) OR ("death"[Title/Abstract])) AND | 70 931 |
| #2 | ((mortality) OR (death))) | [2 169 659](https://pubmed.ncbi.nlm.nih.gov/?term=%28mortality%29+OR+%28death%29&size=100&ac=no&sort=relevance) |
|  | **1 AND 2** | **4 449** |
