# Supplementary material for: Mortality among over 6 million internal and international migrants in Brazil: a study using the 100 Million Brazilian Cohort
Source: Lancet Reg Health Am. 2023 Feb 27;20:100455. doi: 10.1016/j.lana.2023.100455 (PMC9986634; doi:10.1016/j.lana.2023.100455)
Supplement: Translated Summary [file mmc2.docx]

***Editorial Disclaimer:*** *This translation in Portuguese was submitted by the authors and we reproduce it as supplied. It has not been peer reviewed. Our editorial processes have only been applied to the original abstract in English, which should serve as a reference for this manuscript.*

**RESUMO**

**Introdução**: Para compreender se migrantes vivendo em condi**ç**oes de pobreza em países de baixa e média renda (PBMRs) têm menor mortalidade sobre a população não migrante, investigamos os padrões de mortalidade entre migrantes internos e internacionais no Brasil ao longo da vida.

**Métodos**: Vinculamos os dados socioeconômicos da Coorte de 100 Milhões de Brasileiros com dados de mortalidade entre 1º de janeiro de 2011 e 31 de dezembro de 2018. Calculamos as taxas de mortalidade padronizadas por idade por todas as causas e por causas específicas de acordo com o status de migração de homens e mulheres separadamente. Nos usamos modelos de regressão de Cox para estimar as razões de risco de mortalidade (HR) ajustadas por idade e sexo para migrantes internos (ou seja, indivíduos nascidos no Brasil que vivem em um estado diferente de seu nascimento) em comparação com não migrantes nascidos no Brasil; e para migrantes internacionais (ou seja, pessoas nascidas em outro país) em comparação com indivíduos nascidos no Brasil.

**Resultados**: O estudo acompanhou 45.051.476 indivíduos, dos quais 6.057.814 eram migrantes internos e 277.230 eram migrantes internacionais. Os migrantes internos apresentaram mortalidade por todas as causas semelhante à de não migrantes brasileiros (aHR=0,99, IC95%=0,98-0,99), mortalidade marginalmente maior por doenças isquêmicas do coração (aHR=1,04, 95%IC=1·03-1·05) e mortalidade superior para acidente vascular cerebral (aHR=1·11, IC95%=1·09-1·13). Se comparado com pessoas nascidos no Brasil, os migrantes internacionais apresentaram mortalidade por todas as causas 18% menor (aHR=0,82, IC95%=0,80-0,84), com mortalidade até 50% menor por violência interpessoal entre os homens ( aHR=0,50, IC 95%=0,40-0,64), mas maior mortalidade por causas evitáveis ​​relacionadas à saúde materna (aHR=2,17, IC 95%=1,17-4,05).

**Conclusões**: Embora os migrantes internos tenham apresentado mortalidade por todas as causas similar à nao-migrantes, os migrantes internacionais apresentaram menor mortalidade por todas as causas do que não migrantes. Futuras pesquisas usando abordagens interseccionais são necessárias para entender as variações marcantes na mortalidade por status de migração, idade e sexo, e para causas específicas de morte, como elevada mortalidade materna e baixa mortalidade masculina relacionada à violência interpessoal entre migrantes internacionais.

**Financiamento** Wellcome Trust.
